# Risk of cardiac manifestations in adult mitochondrial disease caused by nuclear genetic defects

**DOI:** 10.1136/openhrt-2020-001510

**Published:** 2021-04-01

**Authors:** Albert Zishen Lim, Daniel M Jones, Matthew G D Bates, Andrew M Schaefer, John O'Sullivan, Catherine Feeney, Maria E Farrugia, John P Bourke, Doug M Turnbull, Gráinne S Gorman, Robert McFarland, Yi Shiau Ng

**Affiliations:** 1 Wellcome Centre for Mitochondrial Research, Translational and Clinical Research Institute, Newcastle University, Newcastle upon Tyne, UK; 2 NHS Highly Specialised Service for Rare Mitochondrial Disorders, Newcastle Upon Tyne Hospitals NHS Foundation Trust, Newcastle Upon Tyne, UK; 3 Department of Cardiology, James Cook University Hospital, Middlesbrough, UK; 4 Cardiology, Freeman Hospital Cardiothoracic Centre, Newcastle upon Tyne, UK; 5 Queen Elizabeth University Hospital, Institute of Neurological Sciences, Glasgow, UK

**Keywords:** genetic association studies, genetics, genetic diseases, inborn, epidemiology

## Abstract

**Objective:**

Regular cardiac surveillance is advocated for patients with primary mitochondrial DNA disease. However, there is limited information to guide clinical practice in mitochondrial conditions caused by nuclear DNA defects. We sought to determine the frequency and spectrum of cardiac abnormalities identified in adult mitochondrial disease originated from the nuclear genome.

**Methods:**

Adult patients with a genetically confirmed mitochondrial disease were identified and followed up at the national clinical service for mitochondrial disease in Newcastle upon Tyne, UK (January 2009 to December 2018). Case notes, molecular genetics reports, laboratory data and cardiac investigations, including serial electrocardiograms and echocardiograms, were reviewed.

**Results:**

In this cohort-based observational study, we included 146 adult patients (92 women) (mean age 53.6±18.7 years, 95% CI 50.6 to 56.7) with a mean follow-up duration of 7.9±5.1 years (95% CI 7.0 to 8.8). Eleven different nuclear genotypes were identified: *TWNK*, *POLG, RRM2B, OPA1, GFER*, *YARS2*, *TYMP*, *ETFDH*, *SDHA*, *TRIT1* and *AGK*. Cardiac abnormalities were detected in 14 patients (9.6%). Seven of these patients (4.8%) had early-onset cardiac manifestations: hypertrophic cardiomyopathy required cardiac transplantation (*AGK;* n=2/2), left ventricular (LV) hypertrophy and bifascicular heart block (*GFER;* n=2/3) and mild LV dysfunction (*GFER;* n=1/3, *YARS2;* n=1/2, *TWNK;* n=1/41). The remaining seven patients had acquired heart disease most likely related to conventional cardiovascular risk factors and presented later in life (14.6±12.8 vs 55.1±8.9 years, p<0.0001).

**Conclusions:**

Our findings demonstrate that the risk of cardiac involvement is genotype specific, suggesting that routine cardiac screening is not indicated for most adult patients with nuclear gene-related mitochondrial disease.

**Key questions**

**What is already known about this subject?**

- Approximately one-third of adult patients with mitochondrial disease have nuclear gene inheritance (ie, nuclear gene defect).
- Regular cardiac surveillance is currently advocated for these patients based on the experienced derived from the management of primary mitochondrial DNA disorders.

**What does this study add?**

- In this observational cohort study (n=146), we show that the majority of adult patients with mitochondrial disease of the nuclear genome (~90%) have normal cardiac investigations, and the risk of cardiac involvement is genotype specific.
- Fourteen patients (9.6%) developed cardiac abnormalities, but only half of them (4.8%) were attributed to the mitochondrial dysfunction with a mean follow up of 8 years.

**How might this impact on clinical practice?**

- The risk of cardiac involvement is low in most adult patients with nuclear gene-related mitochondrial disease and we propose a cardiac algorithm to guide clinical practice.

## Introduction

Mitochondrial diseases are a group of neurometabolic disorders that can be caused by genetic defects in both the mitochondrial DNA (mtDNA) and nuclear DNA (nDNA). The estimated prevalence of all forms of adult mitochondrial disease is 1 in 4300, of which approximately a third of patients have nDNA pathogenic variants.^1^ Mitochondrial diseases are clinically heterogeneous and can affect organs with high-energy demand such as central nervous system, skeletal muscle and heart.^2^ The range of cardiac involvement such as hypertrophic cardiomyopathy, conduction abnormalities and sudden cardiac death are commonly reported in patients with certain forms of primary mtDNA disease.^3–6^ However, the spectrum of cardiac manifestation among patients with nDNA defects has only been described in isolated case reports or small case series.^7^ At present, regular cardiac surveillance is routinely recommended for all patients with mitochondrial disease irrespective of the underlying genetic aetiology.^8^

A better understanding of cardiac abnormalities across different genotypes would guide clinicians to tailor cardiac surveillance to high-risk groups and instigate appropriate intervention at the earliest stage. On the other hand, patients with negligible risk of developing a cardiac disease related to the mitochondrial dysfunction can be reassured without requiring regular cardiac screening, potentially reducing the burden of healthcare cost. In this study, we sought to evaluate the cardiac phenotypes systematically in adult patients with mitochondrial disease resulting from pathogenic mutations in the nuclear genes. We also set out to determine the cardiac abnormalities that are attributed to their underlying primary mitochondrial disorders. To progress the current clinical practice towards individualised precision medicine, we aimed to identify patients who were most likely to benefit from regular cardiac screening in this study.

## Methods

### Study design and settings

We identified adult patients harbouring pathogenic nDNA variants from the National Health Service Highly Specialised Service for Rare Mitochondrial Disorders in Newcastle upon Tyne, UK and Mitochondrial Disease Patient Cohort (MitoCohort, UK) (REC: 13/NE/0326), between January 2009 to December 2018. Children who succumbed to fatal cardiac disease had been excluded. These patients were regularly reviewed by clinicians with expertise in mitochondrial disease every 6–24 months (AZL, AMS, GSG, RM, DMT and YSN), and their disease burden, including the degree of cardiac disease severity, was assessed objectively using the Newcastle Mitochondrial Disease Adult Scale, a validated disease rating scale.^9^ Patients had undergone regular cardiac testing (ECG and/or echocardiogram) over a similar period. The abnormal cardiac findings were reviewed by the cardiologists with expertise in mitochondrial diseases, and patients were further assessed if clinically indicated (JPB, MGDB and JO). Demographic data, clinical phenotype, molecular genetic data and blood test results were derived from the MitoCohort. Other medical co-morbidities and drug history were obtained from the clinical notes where available. The majority of genetic diagnoses were achieved via the identification of mitochondrial dysfunction in muscle biopsies (such as large number of cytochrome c oxidase deficient fibres, presence of mtDNA rearrangement/multiple deletions and/or mitochondrial respiratory chain deficiency) and Sanger sequencing of candidates genes based on the clinical phenotype. Our diagnostic algorithm for the mitochondrial disease has been described elsewhere.^10 11^

### Defining cardiac abnormalities in mitochondrial disease

Abnormal echocardiographic findings were reported according to the British Society of Echocardiography and European Society of Cardiology criteria.^12–14^ Left ventricular (LV) dysfunction was categorised into normal (left ventricular ejection fraction (LVEF) >55%), mild (LVEF 45%–55%), moderate (LVEF 35%–44%) and severe (LVEF <35%). LV hypertrophy (LVH) was defined as LV end diastolic septal thickness of >12 mm in adults. Hypertrophic cardiomyopathy was clinically recognised by a maximal LV wall thickness >15 mm in this population. The interpretation of ECG complied with consensus recommendations from professional bodies in clinical cardiology.^15^ Cardiovascular risk factors, as defined by established practice guidelines were ascertained namely, hypertension (persistent blood pressure ≥140/90 mm Hg), diabetes mellitus, hypercholesterolaemia (serum cholesterol level >5 mmol/L), obesity (body mass index >30) and smoking status.^16^ Any cardiac findings with uncertain significance were adjudicated by the cardiologists (JPB, MGDB and JO).

### Patient and public involvement statement

The patients and the public were not involved in the study design, recruitment, statistical analysis and writing of this study.

### Statistical analysis

Statistical analyses were performed using IBM SPSS Statistics (V.24). The incidence rate in this study was expressed as the number of cardiac abnormalities detected per number of years experienced by the cohort at risk in person-years.^17^ Binominal outcome of cardiac investigations from each genotype underwent χ^2^ analysis, including, Fisher’s exact test for counts <5 and parametric data were compared using analysis of variance that had been accounted for normality. Statistical significance was defined as p≤0.05.

## Results

### Clinical phenotypes and genotypes

We identified 146 adults with nDNA defects (92 women) who had complete clinical and cardiac data sets available for the analysis. The mean age of patients was 53.6±18.7 years (95% CI 50.6 to 56.7), and the mean follow-up (FU) duration was 7.9±5.1 years (95% CI 7.0 to 8.8). The common clinical features of mitochondrial disease were eyelid ptosis (54.1%), chronic progressive external ophthalmoplegia (CPEO) (51.4%), cerebellar ataxia (34.2%), myopathy (32.9%), gastrointestinal disturbance (32.2%) and peripheral neuropathy (22.6%) (table 1). Eleven nuclear gene defects were identified: *TWNK* (n=45), *POLG* (n=37), *RRM2B* (n=24), *OPA1* (n=24), *GFER* (n=3), *YARS2* (n=2), *TYMP* (n=3), *ETFDH* (n=2), *SDHA* (n=2), *TRIT1* (n=2) and *AGK* (n=2). Clinical phenotypes associated with individual nuclear defects are summarised in online supplemental table. Three participants died during the FU (POLG—severe epileptic encephalopathy and multiorgan failure, ETFDH—respiratory failure secondary to neuromuscular weakness, TYMP—gastrointestinal sepsis); none of them manifested with any cardiac abnormalities.

10.1136/openhrt-2020-001510.supp1 Supplementary data

**Table 1:** Summary of the patient cohort with Mendelian mitochondrial disease (n=146)

Demographic data	
No of patients	146
Mean age (SD; 95% CI)	53.6 years (18.8; 50.6–56.7)
Mean duration of follow-up (SD; 95% CI)	7.9 years (5.1; 7.0–8.8)
Women (%)	92 (63)
**Clinical features associated with mitochondrial disease**	
Mean age of onset of mitochondrial disease (SD; 95% CI)	29.0 years (19.8; 25.4–32.6)
Eyelid ptosis (%)	79 (54.1)
Progressive external ophthalmoplegia (%)	75 (51.4)
Cerebellar ataxia (%)	50 (34.2)
Myopathy (%)	48 (32.9)
Gastrointestinal disturbance (%)	47 (32.2)
Peripheral neuropathy (%)	33 (22.6)
Epileptic seizures (%)	7 (4.8)
Mean creatine kinase (SD; 95% CI)	216 units/L (218; 171-261)
Mean lactate (SD; 95% CI)	2.2 mmol/L (2.7; 1.6–2.8)
**Cardiovascular risk factors**	
Systolic blood pressure mm Hg (SD; 95% CI)	134 (17.2; 130–138)
Diastolic blood pressure mm Hg (SD; 95% CI)	78 (11.4; 76–81)
Hypertension (%)	36 (4.7)
High cholesterol level >5 mmol/L (%)	29 (19.9)
Positive smoking history (%)	20 (13.7)
Body mass index kg/m^2^ (SD; 95% CI)	26.5 (6.2; 25.1–27.9)
Obesity (BMI >30) (%)	19 (13.0)
Diabetes (%)	16 (11.0)

The cardiovascular risk factors (hypertension, high cholesterol, smoking history, obesity and diabetes) reported were the minimum frequencies of this cohort based on available information.

BMI, body mass index.

### Cardiac abnormalities

**Figure 1:**
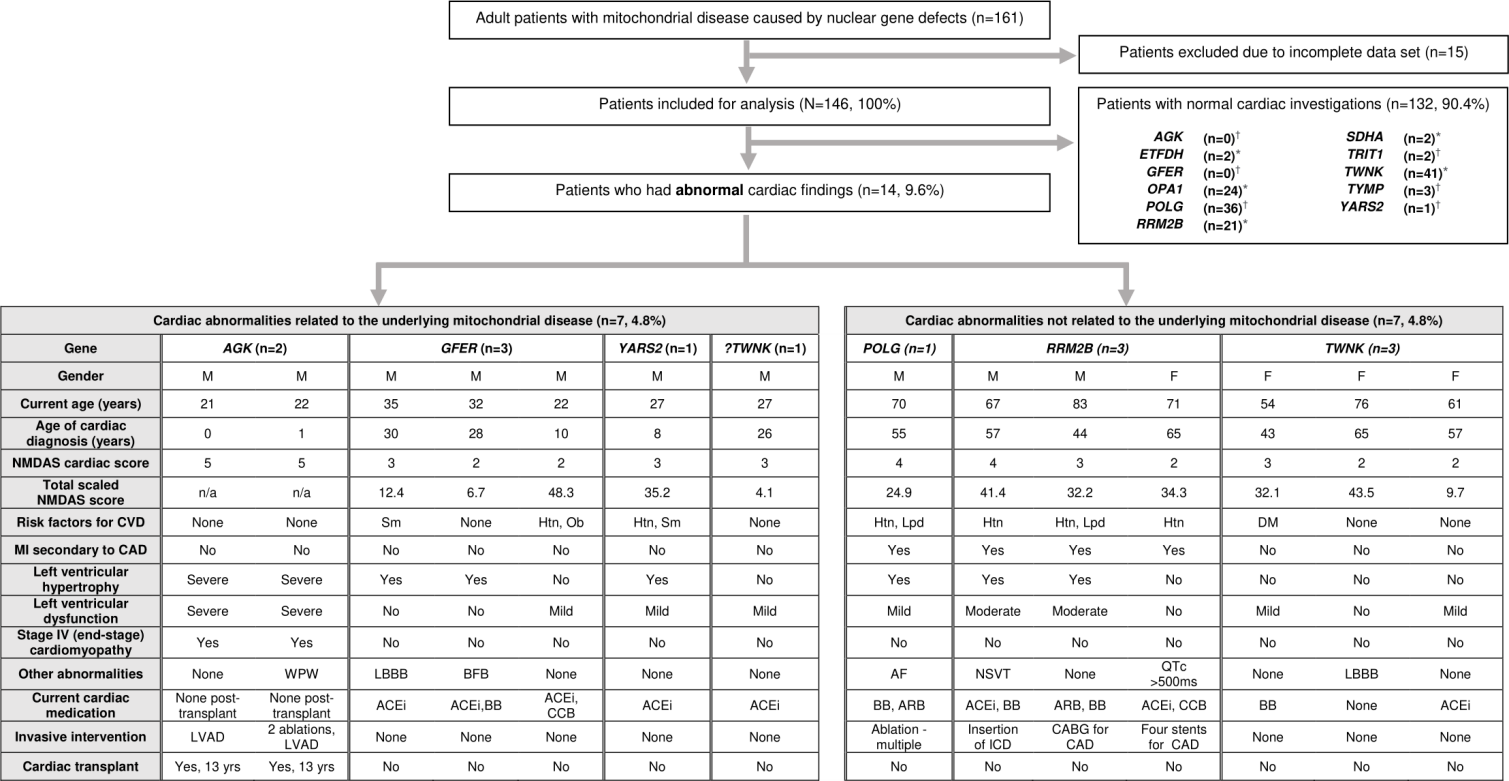
Summary of cardiac abnormalities identified. Left ventricle dysfunction defined as mild (LVEF 45%–55%), moderate (35%–45%) and severe (<35%). NMDAS cardiac score <3 indicates asymptomatic cardiac abnormalities. Other cardiac abnormalities includes conduction and repolarisation conditions. ?=the link between the underlying genetic with cardiac abnormalities is tentative. *The inheritance pattern is autosomal dominant for all cases of *TWNK*, *RRM2B*, *OPA1* and *SDHA* except one *RRM2B* patient who has two pathogenic variants inherited as a recessive disease. †The inheritance pattern is autosomal recessive for the following nDNA defects: *POLG* (except one case with a single heterozygous variant inherited as a dominant disease), *GFER*, *YARS2*, *TYMP*, *ETFDH*, *TRIT1* and *AGK*. AF, atrial fibrillation; ARB, angiotensin receptor blocker; BB, beta-blocker; BFB, bifasicular block; CABG, coronary artery bypass graft; CCB, calcium channel blocker; CAD, coronary artery disease; CM, cardiomyopathy; CVD, cardiovascular disease; DM, diabetes mellitus; Htn, hypertension; ICD, implantable cardioverter defibrillator; Lpd, dyslipidaemia; LVAD, left ventricular assist device; Ob, obesity; NMDAS, Newcastle Mitochondrial Disease Adult Scale; NSVT, non-sustained ventricular tachyarrhythmia; Sm, History of cigarette smoking; WPW, Wolff-Parkinson-White syndrome.

Of the 146 patients with complete clinical and cardiac datasets, the majority (90.4%) had normal cardiac findings during the FU period. We only identified 14 cases with significant cardiac abnormalities (9.6%) (figure 1) with an estimated incidence rate of 1.8 per 1000 person-years (95% CI 1.1 to 3.1). Eight of the 14 patients developed LVH. Reduced LVEF was evident in ten patients: mild (n=7), moderate (n=1) and severe (n=2). Cardiac conduction abnormalities electrical abnormalities were identified in seven (4.8%): Wolff-Parkinson-White (WPW) syndrome (n=1), non-sustained ventricular tachycardia (VT) (n=1), persistent atrial fibrillation (AF) (n=1), left bundle branch block (LBBB) (n=2), bifascicular block (n=1) and asymptomatic QT prolongation (QT*c* >500 ms) (n=1). Coexisting LV dysfunction was identified in the cases of WPW, non-sustained VT and persistent AF (figure 1).

Cardiac abnormalities were attributable to the underlying mitochondrial genotype in seven patients (4.8%): *AGK* (n=2), *GFER* (n=3), *YARS2* (n=1) and *TWNK* (n=1). The mean age of cardiac manifestation was 14.6±12.8 years (95% CI 2.74 to 26.4). Both patients (unrelated) who harboured pathogenic variants in *AGK* presented with an infantile-onset (<1 year old) hypertrophic cardiomyopathy, which progressed to end-stage cardiac failure. Both patients subsequently required LV assist device insertions, followed by orthotopic cardiac transplants at age 13 years. One of the *AGK* cases also had symptomatic WPW syndrome and underwent two accessory pathway (AP) ablation procedures at age 4 years (left lateral AP) and 7 years (left posterolateral AP), respectively.

Two siblings with recessive *GFER* disease were identified to have LVH with preserved ejection fraction, and electrical abnormalities (bifascicular block and LBBB, respectively) in their twenties. The third patient with *GFER* mutation developed mild LV dysfunction without any structural or rhythmic changes at the age of 10 years. A 27-year-old man with *YARS2*-related mitochondrial disease had progressive hypertrophic cardiomyopathy with LV systolic dysfunction atrial dilatation (further details were described elsewhere^18^) that first identified in late childhood. A patient with a heterozygous *TWNK* variant was identified to have mild LV dysfunction when he was investigated for palpitations at the age of 26 years old. Interestingly, his grandmother (77 years old), mother (55 years old) and brother (27 years old) who also harboured the same *TWNK* variant did not exhibit any cardiac involvement.

Seven patients developed cardiac diseases that were deemed unrelated to their genetic defect: *POLG* (n=1), *TWNK* (n=3) and *RRM2B* (n=3). The mean onset of cardiac abnormalities identified in these patients was 55.1±8.9 years (95% CI 46.9 to 63.4). Four patients had had a myocardial infarction secondary to coronary artery disease (*RRM2B*=3; *POLG*=1). The therapeutic interventions for three *RRM2B* patients included emergency coronary angioplasty (n=1), implantable cardioverter defibrillator for prolonged non-sustained VT and moderate LV dysfunction at age 60 years (n=1), and elective coronary artery bypass surgery (n=1). The patient with *POLG*-related mitochondrial disease had persistent AF refractory to multiple ablations and subsequently had an acute admission due to myocardial infarction. Two cases of *TWNK* mutation had asymptomatic, mild LV dysfunction at age 43 and 57 years which remained stable for 11 years and 4 years of FU, respectively. Another *TWNK* case had asymptomatic LBBB detected at the age of 65 years. Overall, the frequencies of cardiac abnormalities within each of these genotypes (*TWNK, POLG* and *RRM2B*) were low even though they were accounted for the two-thirds of adult patients in our cohort. (figure 1).

The frequency of individual cardiovascular risk factors is summarised in table 1. There was no significant association between specific nuclear gene defects and with any of the cardiovascular risk factors. Nineteen patients were treated with an 3-hydroxy-3-methyl-glutaryl-coenzyme A reductase inhibitor (statin); none reported worsening of myopathy, myalgia or other significant adverse side effects. Moreover, there was no significant difference in creatine kinase level between the patients receiving statins (n=19; mean 263.1±249.8 units/L) and patients not receiving statins (n=70; mean 198.9±213 units/L) (p=0.265).

None of the patients with the following genetic defects developed cardiac abnormality during the FU: *OPA1* (mean age 51.9±15.4 years, mean FU 9.2±4.4 years), *TYMP* (mean age 35.0±6.2 years, mean FU 3.7±1.5 years), *ETFDH* (mean age 43.5±17.7 years, mean FU 18.5±10.6 years), *SDHA* (mean age 82±8.5 years, mean FU 17.5±9.2 years) and *TRIT1* (mean age 32.5±5.0 years, mean FU 9.5±2.1 years).

## Discussion

In this observational cohort study of adult patients with mitochondrial disease, we show that the majority of patients with nDNA pathogenic variants have normal cardiac studies. Abnormal cardiac findings were identified in approximately 10% of the patients and half of those with cardiac abnormalities had already presented before adulthood. In contrast, patients with pathogenic mtDNA variants have a significantly higher prevalence of cardiac involvement (25%–50%).^3 4 19^

Our findings of early-onset cardiac involvement caused by pathogenic *AGK* and *YARS2* variants are consistent with the published data.^18 20^ The clinical presentation of two *AGK* cases exhibited classic features of Sengers syndrome^21^ characterised by congenital cataracts, hypertrophic cardiomyopathy, myopathy and exercise intolerance. We previously demonstrated that hypertrophic cardiomyopathy is common among patients who harboured the pathogenic *YARS2* variants, in addition to other clinical findings such as lactic acidosis, proximal myopathy, respiratory muscle weakness and sideroblastic anaemia in some cases.^18^ The exact function of GFER (Growth Factor, Augmenter of Liver Regeneration) protein is not well understood. The *GFER* genetic defect was initially reported in three children who presented with developmental delay, progressive myopathy, congenital cataracts and sensorineural hearing loss.^22^ In comparison, we describe three adult patients with recessive *GFER* variants from two unrelated pedigrees, who developed mild LV dysfunction; thus, expanding the phenotypic spectrum of *GFER*-related mitochondrial disease.

We identified mild LV dysfunction in one patient aged 27 years who harbours a heterozygous *TWNK* variant that was investigated for intermittent palpitations. However, cardiac investigations were normal in three of his family members, who have the same genetic mutation. Non-specific cardiac abnormalities were identified through routine screening in three other *TWNK* patients over the age of 50 years, and they have coexisting cardiovascular risk factors. There is no evidence of cardiac phenotype in the TWINKLE deletor mouse model,^23^ and autosomal dominant *TWNK* mitochondrial disease typically associated with late-onset CPEO and mild myopathy in human. As such, we believe that the risk of early cardiac disease is low in this patient group.^24^

**Figure 2:**
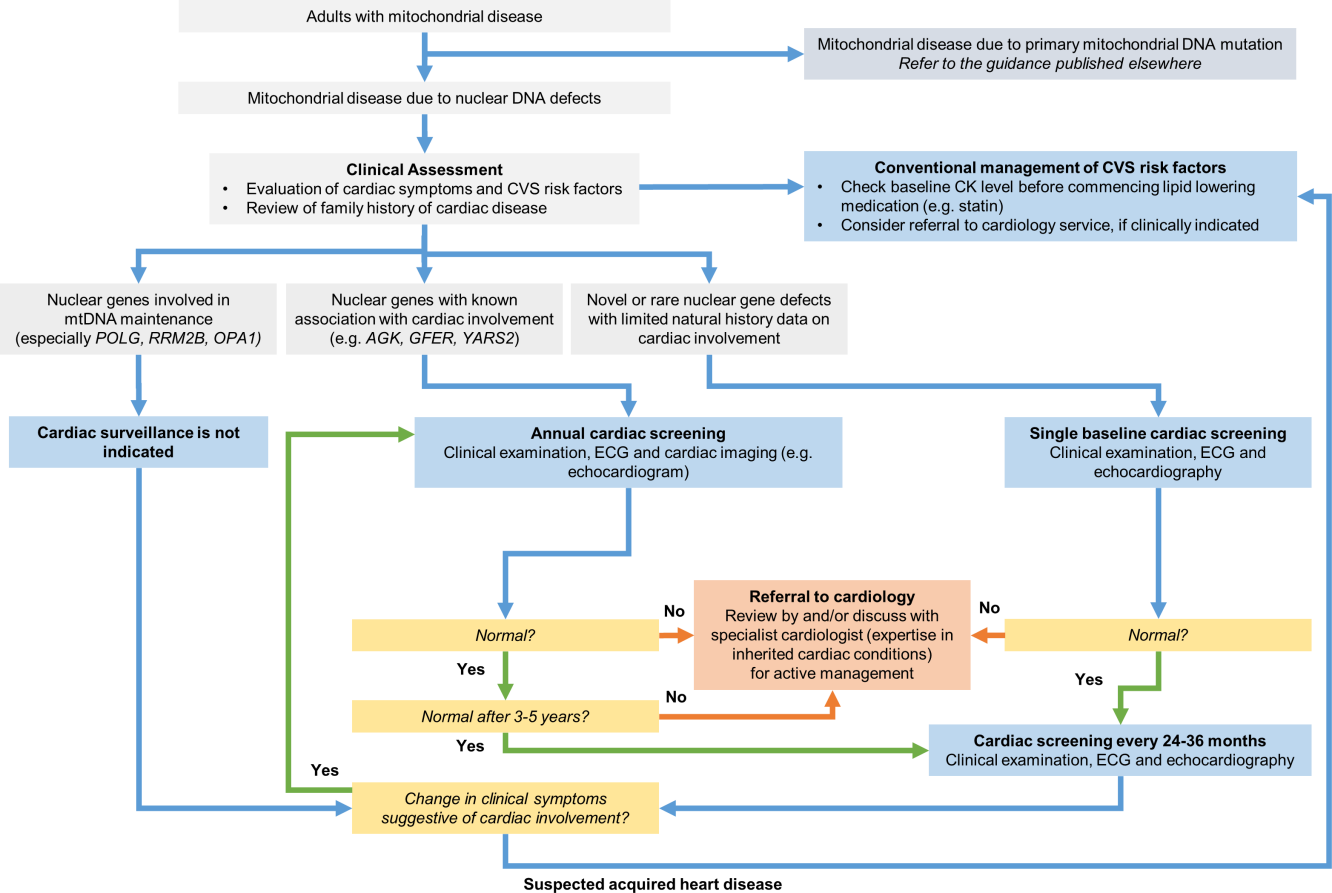
Clinical algorithm to guide cardiac surveillance for the adult mitochondrial disease of nuclear gene defects. This algorithm is proposed guidance and is not intended to replace the clinical judgement of healthcare professionals in the context of individual circumstances. The clinical guidelines for mitochondrial DNA mutations have been published.^3^ Clinical team refers to clinicians who regularly follow-up the patients with mitochondrial disease, and they include general neurologists, clinical geneticists, metabolic medicine physicians and general practitioners/primary care physicians. CK, creatine kinase; CVS, cardiovascular system.

Several nuclear genes involved in the mtDNA maintenance such as *POLG*, *TWNK*, *RRM2B* and *OPA1* account for a substantial proportion of the genetic diagnoses in adult patients. Our results suggest that these common nuclear gene defects do not have an inherent cardiac phenotype apart from a single case of *TWNK* mutation. Based on our longitudinal data, we have proposed a clinical algorithm of cardiac screening for nDNA related mitochondrial disease in adults (figure 2). A baseline cardiac screening is recommended for any novel or ultra rare nuclear gene defects with limited natural history data on their respective cardiac involvement.

A small number of older patients with cardiovascular risk factors developed coronary artery disease similarly seen in the general population, suggesting that the conventional management of cardiovascular risk factors should be instigated accordingly. There is a theoretical concern that using statins could exacerbate myopathy in patients with neuromuscular and mitochondrial diseases.^25 26^ However, we have not observed any significant adverse effect of statin use in our patients.

We have not been able to determine the risk of cardiac disease in rarer nuclear gene defects associated with the mtDNA maintenance such as *MGME1, DNA2, SLC25A4* (also known as *ANT1), DGUOK* and *MPV17*.^27^ The published literature has not revealed that cardiac involvement is a consistent finding in these patients who presented with CPEO and multiple mtDNA deletions on muscle biopsy.^28 29^ However, robust systematic evaluation of the risk of cardiac disease in these genes is warranted to inform clinical practice.

A few recent studies proposed that cardiac surveillance for mitochondrial patients can be stratified based on the clinical syndrome or phenotype alone.^7 8 30^ However, such recommendations have limitations, given that the phenotype–genotype correlation is not always evident. For instance, CPEO and myopathy are common clinical features seen in adult patients with mitochondrial disease and the genetic causes are highly heterogeneous, which include primary mtDNA point mutations such as m.3243A>G, single, large-scale mtDNA deletions or multiple mtDNA deletions secondary to nuclear gene defects as outlined previously.^27 31 32^ If cardiac surveillance were to perform in all patients with CPEO or myopathy, a significant portion of patients with nDNA pathogenic would subject for unnecessary investigations and hospital visits.

There are several limitations in this study. First, we only capture three nuclear genes associated with an early-onset cardiac phenotype in this cohort, and the patients have survived into adulthood. However, there are ultrarare nuclear genetic defects, such as aminoacyl-tRNA synthetase^33^ that can cause fatal cardiac disease in childhood. Second, most of our patients did not have cardiac MR imaging performed. Cardiac MR has a higher sensitivity for the detection of myocardial changes ahead of ventricular dysfunction over routine transthoracic echocardiography.^34 35^ However, the majority of our patients have had serial echocardiograms over long FU to reduce the likelihood of any clinically significant pathology. Third, we only studied the dominant form of mitochondrial disease for these genes: *TWNK*, *RRM2B*, *OPA1* and *SDHA*. We had not recruited patients with the autosomal recessive disease who typically have a more severe phenotype, frequently manifesting in childhood and much less prevalent in the population. Finally, the numbers of patients with specific genotypes (eg, *TRIT1* and *SDHA*) are small in our cohort, and an international, multicentre study would be necessary to establish their risk of cardiac involvement definitively.

## Conclusions

The overall risk of early cardiac involvement in adult mitochondrial disease caused by nuclear gene defects is low (4.8%), and is genotype specific. Our study provides evidence that routine cardiac screening is not indicated for most adult patients with nuclear gene-related mitochondrial disease.

## Data Availability

Data available on request due to privacy/ethical restrictions. Data of deidentified participants are available on request to the oversight committee of UK Mitochondrial Disease Patient Cohort: A Natural History Study and Patient Registry.

## References

[1] Gorman GS, Schaefer AM, Ng Y, et al. Prevalence of nuclear and mitochondrial DNA mutations related to adult mitochondrial disease. Ann Neurol 2015;77:753–9. 10.1002/ana.24362 25652200 PMC4737121

[2] Brunel-Guitton C, Levtova A, Sasarman F. Mitochondrial diseases and cardiomyopathies. Canadian Journal of Cardiology 2015;31:1360–76. 10.1016/j.cjca.2015.08.017 26518446

[3] Bates MGD, Bourke JP, Giordano C, et al. Cardiac involvement in mitochondrial DNA disease: clinical spectrum, diagnosis, and management. Eur Heart J 2012;33:3023–33. 10.1093/eurheartj/ehs275 22936362 PMC3530901

[4] Wahbi K, Bougouin W, Béhin A, et al. Long-term cardiac prognosis and risk stratification in 260 adults presenting with mitochondrial diseases. Eur Heart J 2015;36:2886–93. 10.1093/eurheartj/ehv307 26224072

[5] Limongelli G, Masarone D, Pacileo G. Mitochondrial disease and the heart. Heart 2017;103:390–8. 10.1136/heartjnl-2015-308193 27821705

[6] St-Pierre G, Steinberg C, Dubois M, et al. What the cardiologist should know about mitochondrial cardiomyopathy? Can J Cardiol 2019;35:221–4. 10.1016/j.cjca.2018.11.018 30760430

[7] Quadir A, Pontifex CS, Lee Robertson H, et al. Systematic review and meta-analysis of cardiac involvement in mitochondrial myopathy. Neurol Genet 2019;5:e339. 10.1212/NXG.0000000000000339 31403078 PMC6659349

[8] Parikh S, Goldstein A, Karaa A, et al. Patient care standards for primary mitochondrial disease: a consensus statement from the mitochondrial medicine Society. Genet Med 2017;19. 10.1038/gim.2017.107. [Epub ahead of print: 27 07 2017]. PMC7804217 28749475

[9] Schaefer AM, Phoenix C, Elson JL, et al. Mitochondrial disease in adults: a scale to monitor progression and treatment. Neurology 2006;66:1932–4. 10.1212/01.wnl.0000219759.72195.41 16801664

[10] McFarland R, Taylor RW, Turnbull DM. A neurological perspective on mitochondrial disease. Lancet Neurol 2010;9:829–40. 10.1016/S1474-4422(10)70116-2 20650404

[11] Taylor RW, Pyle A, Griffin H, et al. Use of whole-exome sequencing to determine the genetic basis of multiple mitochondrial respiratory chain complex deficiencies. JAMA 2014;312:68–77. 10.1001/jama.2014.7184 25058219 PMC6558267

[12] Wharton G, Steeds R, Allen J, et al. A minimum dataset for a standard adult transthoracic echocardiogram: a guideline protocol from the British Society of echocardiography. Echo Res Pract 2015;2:G9–24. 10.1530/ERP-14-0079 26693316 PMC4676441

[13] , Elliott PM, Anastasakis A, et al, Authors/Task Force members. 2014 ESC guidelines on diagnosis and management of hypertrophic cardiomyopathy: the Task Force for the Diagnosis and Management of Hypertrophic Cardiomyopathy of the European Society of Cardiology (ESC). Eur Heart J 2014;35:2733–79. 10.1093/eurheartj/ehu284 25173338

[14] Douglas PS, Carabello BA, Lang RM, et al. 2019 ACC/AHA/ASE key data elements and definitions for transthoracic echocardiography: a report of the American College of Cardiology/American heart association Task force on clinical data standards (writing Committee to develop cardiovascular endpoints data standards) and the American Society of echocardiography. Circ Cardiovasc Imaging 2019;12:e000027. 10.1161/HCI.0000000000000027 31233331

[15] Kligfield P, Gettes LS, Bailey JJ, et al. Recommendations for the standardization and interpretation of the electrocardiogram. Part I: the electrocardiogram and its technology. A scientific statement from the American heart association electrocardiography and arrhythmias Committee, Council on clinical cardiology; the American College of cardiology Foundation; and the heart rhythm Society. Heart Rhythm 2007;4:394–412. 10.1016/j.hrthm.2007.01.027 17341413

[16] Goff DC, Lloyd-Jones DM, Bennett G, et al. 2013 ACC/AHA guideline on the assessment of cardiovascular risk: a report of the American College of Cardiology/American Heart Association Task Force on Practice Guidelines. Circulation 2014;129:S49–73. 10.1161/01.cir.0000437741.48606.98 24222018

[17] Rothman KJ. Epidemiology: an introduction. Oxford university press, 2012.

[18] Sommerville EW, Ng YS, Alston CL, et al. Clinical features, molecular heterogeneity, and prognostic implications in YARS2-related mitochondrial myopathy. JAMA Neurol 2017;74:686–94. 10.1001/jamaneurol.2016.4357 28395030 PMC5822212

[19] Hollingsworth KG, Gorman GS, Trenell MI, et al. Cardiomyopathy is common in patients with the mitochondrial DNA m.3243A>G mutation and correlates with mutation load. Neuromuscul Disord 2012;22:592–6. 10.1016/j.nmd.2012.03.001 22513320 PMC3387369

[20] Haghighi A, Haack TB, Atiq M, et al. Sengers syndrome: six novel AGK mutations in seven new families and review of the phenotypic and mutational spectrum of 29 patients. Orphanet J Rare Dis 2014;9:1–12. 10.1186/s13023-014-0119-3 25208612 PMC4167147

[21] Morava E, Sengers R, Ter Laak H, et al. Congenital hypertrophic cardiomyopathy, cataract, mitochondrial myopathy and defective oxidative phosphorylation in two siblings with Sengers-like syndrome. Eur J Pediatr 2004;163:467–71. 10.1007/s00431-004-1465-2 15168109

[22] Di Fonzo A, Ronchi D, Lodi T, et al. The mitochondrial disulfide relay system protein GFER is mutated in autosomal-recessive myopathy with cataract and combined respiratory-chain deficiency. Am J Hum Genet 2009;84:594–604. 10.1016/j.ajhg.2009.04.004 19409522 PMC2681006

[23] Tyynismaa H, Mjosund KP, Wanrooij S, et al. Mutant mitochondrial helicase Twinkle causes multiple mtDNA deletions and a late-onset mitochondrial disease in mice. Proc Natl Acad Sci U S A 2005;102:17687–92. 10.1073/pnas.0505551102 16301523 PMC1308896

[24] Fratter C, Gorman GS, Stewart JD, et al. The clinical, histochemical, and molecular spectrum of PEO1 (Twinkle)-linked adPEO. Neurology 2010;74:1619–26. 10.1212/WNL.0b013e3181df099f 20479361 PMC2875130

[25] Apostolopoulou M, Corsini A, Roden M. The role of mitochondria in statin-induced myopathy. Eur J Clin Invest 2015;45:745–54. 10.1111/eci.12461 25991405

[26] Mancini GBJ, Baker S, Bergeron J, et al. Diagnosis, prevention, and management of statin adverse effects and intolerance: Canadian consensus Working group update (2016). Canadian Journal of Cardiology 2016;32:S35–65. 10.1016/j.cjca.2016.01.003 27342697

[27] Sommerville EW, Chinnery PF, Gorman GS, et al. Adult-onset Mendelian PEO associated with mitochondrial disease. J Neuromuscul Dis 2014;1:119–33. 10.3233/JND-140041 27858775

[28] El-Hattab AW, Scaglia F. Mitochondrial DNA depletion syndromes: review and updates of genetic basis, manifestations, and therapeutic options. Neurotherapeutics 2013;10:186–98. 10.1007/s13311-013-0177-6 23385875 PMC3625391

[29] Viscomi C, Zeviani M. MtDNA-maintenance defects: syndromes and genes. J Inherit Metab Dis 2017;40:587–99. 10.1007/s10545-017-0027-5 28324239 PMC5500664

[30] Pfeffer G, Mezei MM. Cardiac screening investigations in adult-onset progressive external ophthalmoplegia patients. Muscle Nerve 2012;46:593–6. 10.1002/mus.23538 22987704

[31] Grady JP, Campbell G, Ratnaike T, et al. Disease progression in patients with single, large-scale mitochondrial DNA deletions. Brain 2014;137:323–34. 10.1093/brain/awt321 24277717 PMC3914470

[32] Orsucci D, Angelini C, Bertini E, et al. Revisiting mitochondrial ocular myopathies: a study from the Italian network. J Neurol 2017;264:1777–84. 10.1007/s00415-017-8567-z 28695364

[33] Sissler M, González-Serrano LE, Westhof E. Recent advances in mitochondrial aminoacyl-tRNA synthetases and disease. Trends Mol Med 2017;23:693–708. 10.1016/j.molmed.2017.06.002 28716624

[34] Marwick TH, Neubauer S, Petersen SE. Use of cardiac magnetic resonance and echocardiography in population-based studies: why, where, and when? Circ Cardiovasc Imaging 2013;6:590–6. 10.1161/CIRCIMAGING.113.000498 23861451

[35] Bates MGD, Hollingsworth KG, Newman JH, et al. Concentric hypertrophic remodelling and subendocardial dysfunction in mitochondrial DNA point mutation carriers. Eur Heart J Cardiovasc Imaging 2013;14:650–8. 10.1093/ehjci/jes226 23129433 PMC3681541

