# Supplementary material for: Risk of cardiac manifestations in adult mitochondrial disease caused by nuclear genetic defects
Source: Open Heart. 2021 Apr 1;8(1):e001510. doi: 10.1136/openhrt-2020-001510 (PMC8021886; doi:10.1136/openhrt-2020-001510)
Supplement: Supplementary data [file openhrt-2020-001510supp001.pdf]

Gene	OMIM	Inheritance pattern	Function of gene	Clinical features
AGK	610345	AR	Acylgycerol kinase is a mitochondrial membrane protein, a lipid kinase that phosphorylate monoacylglycerol and diacylglycerol to form lysophosphatidic acid and phosphatidic acid, respectively.	Sengers syndrome, congenital cataracts, hypertrophic cardiomyopathy, skeletal myopathy, exercise intolerance and lactic acidosis.
ETFDH	231675	AR	Electron transfer flavoprotein dehydrogenase, an enzyme involved in the electron transfer from at least 9 mitochondrial flavin-containing dehydrogenases to the main mitochondrial respiratory chain	Glutaric acidaemia type II, multiple acyl-CoA dehydrogenation deficiency (MADD), riboflavin-responsive encephalopathy/ myopathy, metabolic acidosis, hypoglycaemia, brain malformations and hepatomegaly
GFER	600924	AR	Growth Factor, ERV1-like is a FAD-dependent sulfhydryl oxidase that regenerates the redox-active disulfide bonds in CHCHD4/MIA40, a chaperone for disulfide bond formation and protein folding in the mitochondria.	Progressive myopathy, congenital cataracts, sensori-neural hearing loss, developmental delay and multiple respiratory-chain deficiency
OPA1	605290	AD	Mitochondrial dynamin like GTPase is a protein located on the inner mitochondrial membrane that regulates the stability of the mitochondrial network and the sequestration of proapoptotic cytochrome c oxidase molecules.	Dominant optic atrophy and some patients may develop additional features such as hearing loss, CPEO, ataxia, peripheral neuropathy, myopathy and Behr syndrome
POLG	174763	AD/AR*	Polymerase gamma (pol γ) repairs and replicates mitochondrial DNA.	Alpers-Huttenlocher syndrome, ataxia neuropathy spectrum, sensory ataxia, neuropathy, dysarthria, CPEO and rarely childhood myocerebrohepatopathy syndrome.
RRM2B	604712	AD/AR*	Ribonucleotide reductase regulatory TP53 inducible subunit M2B, makes p53 inducible small subunit, a protein that helps to produce nucleotides that form the mitochondrial DNA and to maintain a normal amount of DNA in cells.	Mitochondrial DNA depletion syndrome characterised by encephalomyopathic manifestation with renal tubulopathy, sensorineural hearing loss and myopathy. Adult patients with dominant disease usually present with CPEO and myopathy.
SDHA	600857	AD/AR	Succinate dehydrogenase complex flavoprotein subunit A is one of the subunits of mitochondrial complex II enzyme. It links two pathways in energy conversion (citric acid cycle and oxidative phosphorylation). SDH enzyme converts succinate to fumarate.	Leigh syndrome characterised by progressive loss of mental and movement abilities in infancy or childhood in patients with recessive mutations. Some patients may present with muscle weakness, movement disorders, seizures and poor bulbar function.
TRIT1	617840	AR	tRNA isopentenyltransferase 1 modifies transfer RNAs by adding a dimethylallyl group onto the adenine at position 37. This modification maintains the correct reading frame during protein translation.	Global developmental delay, intellectual disability, microcephaly, diabetes mellitus and early-onset seizures.
TWNK	606075	AD/AR*	Twinkle mtDNA helicase plays a crucial role on the replication of mtDNA by unwinding the double-stranded DNA.	Recessive mutations cause infantile-onset spinocerebellar ataxia. Perrault syndrome has been described (hearing loss and ovarian insufficiency in affected females). Adult patients with dominant disease usually present with indolent CPEO and myopathy.
TYMP	131222	AR	Thymidine phosphorylase converts thymidine into 2-deoxyribose 1-phosphate and thymine. This chemical reaction is an important step in the breakdown of thymidine, which helps regulate the level of nucleosides in cells.	Mitochondrial neurogastrointestinal encephalopathy (MNGIE) syndrome characterised by leukoencephalopathy, peripheral neuropathy, CPEO, hearing loss and pseudo-obstruction.
YARS2	610957	AR	Tyrosyl-tRNA synthetase 2 catalyses the attachment of tyrosine (Tyr) to the tRNA (Tyr).	Myopathy with lactic acidosis, sideroblastic anaemia (MLASA) syndrome or myopathy and cardiomyopathy only

Supplementary Table. Overview of the gene function, inheritance pattern and clinical phenotype in nDNA-related mitochondrial diseases presented in the manuscript. The inheritance patterns in some of the mitochondrial diseases can be either autosomal recessive or autosomal dominant. *denotes autosomal recessive inheritance that is associated with mitochondrial DNA depletion (reduction of the mtDNA copy number) and more severe disease of central nervous system compared to their dominant counterparts, which are associated with multiple mtDNA deletions, chronic progressive external ophthalmoplegia and myopathy. AD – Autosomal dominant, AR – Autosomal recessive, CPEO= chronic progressive external ophthalmoplegia, OMIM – Online Mendelian Inheritance in Man.
